# Does early extubation after cardiac surgery lead to a reduction in intensive care unit length of stay?

**DOI:** 10.1093/icvts/ivac008

**Published:** 2022-02-10

**Authors:** Marcus Taylor, Denish Apparau, Roberto Mosca, Nnamdi Nwaejike

**Affiliations:** 1 Department of Cardiothoracic Surgery, Manchester University Hospital NHS Foundation Trust, Wythenshawe Hospital, Manchester, UK; 2 Department of Cardiothoracic Anaesthesia, Manchester University Hospital NHS Foundation Trust, Wythenshawe Hospital, Manchester, UK

**Keywords:** Cardiac surgery, Fast track, Extubation

## Abstract

A best evidence topic in cardiac surgery was written according to a structured protocol. The question addressed was whether early extubation (EE) after cardiac surgery leads to a reduction in intensive care unit (ICU) length of stay (LOS)? A total of 564 papers were found using the reported search, of which 4 were randomized trials and hence represented the best evidence to answer the clinical question. The authors, journal, date and country of publication, patient group studied, study type, relevant outcomes and results of these papers are tabulated. EE was defined as extubation in theatre (*n* = 2), within 6 h of surgery (*n* = 1) and within 8 h of surgery (*n* = 1). EE was associated with significantly reduced ICU LOS in all studies. Despite the Society of Thoracic Surgeons using extubation <6 h after surgery as a measure of quality, this study has demonstrated that no standardized definition for EE currently exists. The body of evidence identified in this work has demonstrated that for appropriately selected patients (avoiding patients with multiple comorbidities, advanced age and undergoing complex non-elective surgery) early tracheal extubation is associated with a reduction in ICU LOS without an increase in the rate of postoperative complications.

## INTRODUCTION

A best evidence topic was constructed according to a structured protocol. This is fully described in the *ICVTS* [1].

## THREE-PART QUESTION

In [adult patients undergoing cardiac surgery], does [early extubation] lead to [a reduction in Intensive Care Unit length of stay]?

## CLINICAL SCENARIO

You are an adult cardiac surgeon who suggests to colleagues that early extubation (EE) of patients would lead to reduced intensive care unit (ICU) length of stay (LOS). Your colleagues are not convinced, and you therefore review the literature to identify any randomized trials performed, which will help inform your discussion.

## SEARCH STRATEGY

A literature search was undertaken using PubMed from 01 January 2000 to 31 January 2021 to identify articles for inclusion. The employed search strategy was: [extubation OR extubate] AND [cardiac surgery OR cardiac procedures] AND [early OR rapid OR fast-track OR fast track]. The search was limited to human adult subjects and the English language.

## SEARCH OUTCOME

A total of 564 papers were found using the reported search. All abstracts were screened by 2 reviewers (Marcus Taylor and Denish Apparau) and all potentially relevant studies were subsequently reviewed in full by the same 2 reviewers. Any disagreements regarding article selection were resolved by discussion with an additional reviewer (Nnamdi Nwaejike). All non-randomized and retrospective studies were excluded. We included all comparative randomized trial papers, which provided ICU LOS data for EE versus non-EE adult patients undergoing cardiac surgery. To include only papers relevant to contemporary practice, all studies published prior to 2000 were also excluded. Only studies where the extubation strategy differed between groups were included. After screening all relevant papers, 4 papers were identified that provided the best evidence to answer the question. These are presented in Table 1.

**Table 1:** Best evidence papers

Author, date, journal and country Study type (level of evidence)	Patient group	Outcomes	Outcomes and key results	Comments
Totonchi *et al*. (2018), Anesth Pain Med, Iran [2] Single-centre RCT (level II)	100 patients Elective CABG, valve surgery or ASD closure in patients aged 18–65 with BMI 18–25 kg/m^2^ and LVEF >35% Group 1 (extubation in theatre): *n* = 50 Group 2 (ICU extubation): *n* = 50	Median ICU stay Mean drainage during first 24 h Mean CPB time Mean cross-clamp time	Group 1: 34 h (IQR 21.5–44) Group 2: 48 h (IQR 44–60) *P* < 0.001 Group 1: 243.5 ml (±SD 137.9) Group 2: 551.8 ml (±SD 326.1) *P* = 0.001 Group 1: 57.04 min (±SD 23.05) Group 2: 54.97 min (±SD 32.20) *P* = 0.736 Group 1: 39.62 min (±SD 13.79) Group 2: 46.58 min (±SD 12.48) *P* = 0.014	No cases of reintubation occurred 2 patients not extubated in theatre
Salah *et al.* (2015), Heart Lung Vessel, Egypt [3] Single-centre RCT (level II)	52 patients All elective cardiac surgery Group 1 (extubation in theatre): *n* = 26 Group 2 (extubation in ICU): *n* = 26	Mean ICU stay Mean CPB time Mean cross-clamp time Bleeding Reopening Reintubation	Group 1: 57.4 h (±SD 18.6) Group 2: 95.0 h (±SD 33.6) *P* < 0.001 Group 1: 70.15 min (±SD 23.05) Group 2: 86.35 min (±SD 12.13) *P* = 0.003 Group 1: 49.81 min (±SD 19.31) Group 2: 61.92 min (±SD 11.58) *P* = 0.008 Group 1: 34.6% (*n* = 9) Group 2: 0.0% (*n* = 0) *P* = 0.002 Group 1: 11.5% (*n* = 3) Group 2: 0.0% (*n* = 0) *P* = 0.235 Group 1: 7.7% (*n* = 2) Group 2: 0.0% (*n* = 0) *P* = 0.490	1 patient not extubated in theatre
Probst *et al*. (2014), Crit Care, Germany [4] Single-centre RCT (level II)	200 patients Elective CABG and/or valve Group 1 (extubation <6 h after surgery): *n* = 100 Group 2 (extubation >6 h after surgery): *n* = 100	Median ICU stay Median CPB time Median cross-clamp time Reoperation Reintubation	Group 1: 3.3 h (IQR 2.7–4.0) Group 2: 17.9 h (IQR 10.3–24.9) *P* < 0.001 Group 1: 100 min (IQR 75–127) Group 2: 99 min (IQR 79–122) *P* = 0.910 Group 1: 64 min (IQR 51–79) Group 2: 66 min (IQR 51–80) *P* = 0.690 Group 1: 5% (*n* = 5) Group 2: 11% (*n* = 11) *P* = 0.190 Group 1: 5% (*n* = 5) Group 2: 10% (*n* = 10) *P* = 0.280	3 patients not extubated within 6 h of surgery
Simeone *et al.* (2002), J Cardiovasc Surg, Italy [5] Single-centre RCT (level II)	49 patients Elective CABG or valve surgery Group 1 (extubation <8 h after surgery): *n* = 24 Group 2 (extubation >8 h after surgery): *n* = 25	Mean ICU stay Mean CPB time Mean cross-clamp time Reoperation Reintubation	Group 1: 29.0 h (±SD 15.8) Group 2: 46.1 h (±SD 33.9) *P* = 0.030 Group 1: 98.0 min (±SD 32.1) Group 2: 113.7 min (±SD 30.3) *P* > 0.005 Group 1: 75.2 min (±SD 26.6) Group 2: 79.6 min (±SD 22.6) *P* > 0.005 Group 1: 5% (*n* = 5) Group 2: 11% (*n* = 11) *P* = 0.190 Group 1: 5% (*n* = 5) Group 2: 10% (*n* = 10) *P* = 0.280	Rate of successful early extubation not reported Postoperative complication rates not reported

ASD: atrial septal defect; BMI: body mass index; CABG: coronary artery bypass grafting; CPB: cardiopulmonary bypass; ICU: intensive care unit; IQR: interquartile range; LVEF: left ventricular ejection fraction; RCT: randomized control trial; SD: standard deviation.

## RESULTS

The 4 studies ranged in size from 49–200 patients included. All were single-centre randomized trials. Reported outcome metrics included mean [± standard deviation (SD)] ICU stay (*n* = 2) and median [± interquartile range (IQR)] ICU stay (*n* = 2), with all studies reporting outcomes measured in hours. Amongst the studies, EE was defined in 3 different ways. These included extubation in theatre (*n* = 2), within 6 h of surgery (*n* = 1) and within 8 h of surgery (*n* = 1).

Totonchi *et al.* [2] included 100 patients aged 18–65 with left ventricular ejection fraction >35% and body mass index 18–25 kg/m^2^ undergoing elective on-pump coronary artery bypass grafting (CABG), valve surgery or atrial septal defect closure prior to 2018. The 2 groups were well matched in terms of preoperative characteristics. Whilst the EE group had a significantly shorter cross-clamp time, there was no significant difference between groups with regard to cardiopulmonary bypass times. The median ICU stay was significantly reduced for patients extubated in theatre [34 h (± IQR 21.5–44) vs 48 h (± IQR 44–60), *P* < 0.001]. In total, 96.0% (*n* = 48) of the patients in the EE group were extubated in theatre. Drainage in the first 24 h was significantly lower for the EE group but was not significantly different between groups for the subsequent 24 h. There were no cases of reintubation. No other complications were detailed in the study.

Salah *et al.* [3] included 52 patients undergoing all elective cardiac surgery procedures between 2011 and 2013. The mean ICU stay was significantly reduced for patients extubated in theatre [57.4 h (± SD 18.6) vs 95.0 h (± SD 33.6), *P* < 0.001]. However, the groups were not well matched: patients in the EE group had significantly fewer comorbidities, significantly higher mean left ventricular ejection fraction and significantly shorter cardiopulmonary bypass and cross-clamp times. Only 1 patient in the EE group was not extubated in theatre. Whilst the rate of postoperative bleeding was significantly higher in the EE group, the rate of postoperative myocardial ischaemia was significantly lower. The rates of all other complications, including reintubation, did not differ significantly between groups.

Probst *et al.* [4] included 200 patients undergoing elective on-pump CABG and/or valve surgery or atrial septal defect closure prior to 2014. The 2 groups were well matched in terms of pre- and intraoperative characteristics. The median ICU stay was significantly shorter for patients extubated within 6 h of surgery [3.3 h (± IQR 2.7–4.0) vs 17.9 h (± IQR 10.3–24.9), *P* < 0.001]. In total, 97.0% (*n* = 97) of the patients in the EE group were extubated within 6 h of surgery. The rate of cardiac arrhythmia, prolonged respiratory insufficiency and need for cardiopulmonary resuscitation was significantly lower for the EE group. The rates of all other complications, including reintubation, did not differ significantly between groups. There was also no significant difference in overall hospital LOS between the 2 groups (*P* = 0.42).

Simeone *et al.* [5] included 49 patients undergoing elective on-pump CABG or valve surgery between February and November 1999. The 2 groups were well matched in terms of pre- and intraoperative characteristics. The mean ICU stay was significantly reduced for patients extubated within 8 h of surgery [29.0 h (±SD 15.8) vs 46.1 h (±SD 33.9), *P* < 0.001]. The proportion of patients successfully undergoing EE was not reported. In addition, no formal comparison of the rate of complications between groups was presented.

## CLINICAL BOTTOM LINE

Despite the Society of Thoracic Surgeons using extubation <6 h after surgery as a measure of quality [6], this study has demonstrated that no standardized definition for EE currently exists. The body of evidence identified in this work has demonstrated that for appropriately selected patients (avoiding patients with multiple comorbidities, advanced age and undergoing complex non-elective surgery) early tracheal extubation is associated with a reduction in ICU LOS without an increase in the rate of postoperative complications.

**Conflict of interest:** none declared. 

### Reviewer information

Interactive CardioVascular and Thoracic Surgery thanks Alexander Kogan, Roman Gottardi and the other, anonymous reviewer(s) for their contribution to the peer review process of this article.

## References

[1] Dunning J , Prendergast B, Mackway JK. Towards evidence-based medicine in cardiothoracic surgery: best BETS. Interact CardioVasc Thorac Surg 2003;2:405–9. 17670084 10.1016/S1569-9293(03)00191-9

[2] Totonchi Z , Azarfarin R, Jafari L, Ghavidel A, Baharestani B, Alizadehasi A, et al Feasibility of on-table extubation after cardiac surgery with cardiopulmonary bypass: a randomized clinical trial. Anesthesiol Pain Med 2018;8:80158. 10.5812/aapm.80158 PMC6240920 30533392

[3] Salah M , Hosny H, Salah M, Saad H. Impact of immediate versus delayed tracheal extubation on length of ICU stay of cardiac surgical patients, a randomized trial. Hear Lung Vessel 2015;7:311–9. PMC4712034 26811837

[4] Probst S , Cech C, Haentschel D, Scholz M, Ender J. A specialized post-anaesthetic care unit improves fast-track management in cardiac surgery: a prospective randomized trial. Crit Care 2014;18:468. 25123092 10.1186/s13054-014-0468-2 PMC4243831

[5] Simeone F , Biagioli B, Scolletta S, Marullo A, Marchet-Ti L, Caciorgna M, et al Optimization of mechanical ventilation support following cardiac surgery. J Cardiovasc Surg 2002;43:633–41. 12386574

[6] Goeddel LA , Hollander KN, Evans AS. Early extubation after cardiac surgery: a better predictor of outcome than metric of quality? J Cardiothorac Vasc Anesth 2017;32:745–7. 10.1053/j.jvca.2017.12.037 29395821

